# Cohort profile: understanding health service system needs for people with intellectual disability using linked data in New South Wales, Australia

**DOI:** 10.4178/epih.e2024054

**Published:** 2024-06-12

**Authors:** Simone Reppermund, Preeyaporn Srasuebkul, Claire M. Vajdic, Sallie-Anne Pearson, Rachael E. Moorin, Julian N. Trollor

**Affiliations:** 1 Department of Developmental Disability Neuropsychiatry, Discipline of Psychiatry and Mental Health, School of Clinical Medicine, Faculty of Medicine and Health, University of New South Wales, Sydney, Australia; 2 Centre for Healthy Brain Ageing, Discipline of Psychiatry and Mental Health, School of Clinical Medicine, Faculty of Medicine and Health, University of New South Wales, Sydney, Australia; 3 Centre for Big Data Research in Health, Faculty of Medicine and Health, University of New South Wales, Sydney, Australia; 4 Kirby Institute, University of New South Wales, Sydney, Australia; 5 School of Population Health, Faculty of Medicine and Health, University of New South Wales, Sydney, Australia; 6 School of Population Health, Curtin University, Perth, Australia; 7 School of Population and Global Health, The University of Western Australia, Perth, Australia

**Keywords:** Data linkage, Intellectual disability, Health service

## Abstract

This cohort profile describes one of the largest linked datasets in the world concerning the health of people with intellectual disability. The cohort comprises a retrospective group of 100,089 individuals with intellectual disability who received disability and/or health services in New South Wales, Australia. Of these participants, 34% were female, with a median age at cohort entry of 3 years (interquartile range, 0-19). A separate comparator cohort included 455,677 individuals, matched by 5-year age group, sex, and residential postcode at a 5:1 ratio. Initial results indicate that between 2001 and 2018, people with intellectual disability experienced more than double the rate of hospitalisations (538 vs. 235 per 1,000 person-years), as well as markedly higher rates of emergency department presentations (707 vs. 379 per 1,000 person-years) and use of ambulatory mental health services (1,012 vs. 157 per 1,000 person-years), relative to the comparator cohort. The largest disparities in hospital admissions were for mental disorders, dialysis, and diseases of the nervous system and sense organs. Furthermore, individuals with intellectual disability had more than double the rate of dispensed medications found in the comparator cohort. Of these medications, 46.6% were for the treatment of nervous system conditions, as opposed to 24.7% for the comparator cohort. The mean±standard deviation age at death was 52±19 years for people with intellectual disability and 64±22 years for the comparator participants.

## INTRODUCTION

### Intellectual disability and health

Intellectual disability is characterised by impairments in general cognitive abilities that become apparent during the developmental period and substantially impact a person’s adaptive functioning [1]. Approximately 1% of individuals have an intellectual disability [2]. This population experiences extremely poor health [3,4] and faces multiple barriers to accessing timely, affordable, and appropriately equipped health services [5]. Across the lifespan, estimates of the prevalence of mental health conditions in people with intellectual disability range from 28% [6] to 38% [7]. Additionally, these individuals are affected by a wide array of physical health conditions, including a high prevalence of several chronic diseases [8] (refer to [4] for a comprehensive review). The disparity between the health needs of people with intellectual disability and the availability of accessible services has serious consequences, as evidenced by markedly higher mortality rates compared to the general population. The odds ratios for mortality vary from 2.86 to 13.15, depending on the severity of the intellectual disability [9]. The leading causes of death in this group are respiratory, circulatory, and nervous system diseases, as well as cancers. The proportion of deaths from preventable causes is around twice that of the general population [10]. Individuals with intellectual disability face an increased risk of several potentially preventable diseases [4] and have a lower life expectancy than the general population [11-14]. These findings are consistent internationally, although most research has been conducted in Europe, North America and Oceania [4,15].

#### Medicine use and prescribing patterns

In addition to poor health, people with intellectual disability may be prescribed medicines that contribute to adverse health outcomes. This population is systematically excluded from clinical trials on medication use, leaving a gap in the evidence base [16], and inappropriate polypharmacy is widespread [14-17]. Australian survey data have revealed that polypharmacy affects 21% of this demographic [17]. An Irish cross-sectional study reported that within their nationally representative sample of adults over 40 years old, 35% were taking between 5 and 9 medications, and 21% were taking 10 or more [18]. Notably, the prescription of psychotropic medications is disproportionately high among people with intellectual disability, with a rate nearly twice that seen in the general population [19-21]. Antipsychotics are the most frequently prescribed psychotropics within this group [19,22] and are often administered for challenging behaviours, a practice not generally supported by evidence [21]. Research has demonstrated that up to 71% of antipsychotic prescriptions in primary care are issued to individuals with intellectual disability in the absence of a severe mental illness [20]. The use of psychotropic medications in the general population has been linked to increased risk of cardiometabolic morbidity and mortality [23], which may contribute to the poor health outcomes observed in people with intellectual disability. With this cohort study, we can investigate patterns of prescribing, the prevalence of polypharmacy, gaps in prescriptions for preventive healthcare, and the relationship between prescribing patterns and health outcomes over time.

#### Role of primary care

Primary care represents the cornerstone of accessible healthcare for individuals with intellectual disability. It offers an initial point of contact with the healthcare system and pathways to further services, predominantly through general practitioners (GPs). GPs are pivotal in delivering preventive health initiatives, such as screenings, to reduce morbidity and mortality [24,25]. However, the literature includes no large-scale Australian longitudinal data on the health services provided to people with intellectual disability by GPs or how these services compare to those received by people without intellectual disability [25]. Large-scale population-based studies are needed to characterise the health status of people with intellectual disability. These studies should include a detailed examination of patterns of morbidity, comorbidity, GP visits, specialist referrals, allied health consultations, prescription medicine use, and preventive health screening, in comparison with the general population. Such research may shed light on potentially modifiable risk factors and underpin the development of tailored interventions for people with intellectual disability.

### Surveys versus data linkage

Surveys are the most common methodology used to estimate disease prevalence among people with intellectual disability [26-28]. These typically depend on proxy or self-reports, and not all previous studies have included appropriate comparison groups. Moreover, individuals with intellectual disability are not routinely systematically identified or included in general population health surveys. In Australia, large-scale studies characterising health profiles, health resource utilisation, and their determinants in people with intellectual disability, relative to the general population, are scarce. Furthermore, existing population-based studies frequently lack data on prescribed medications and primary care [29,30].

Data linkage represents an effective method for profiling the health service usage of individuals with intellectual disability. Given Australia’s dispersed and relatively small population, the most efficient means of thoroughly characterising the health of this demographic is through multi-jurisdictional linked administrative and specialist data, provided these datasets can adequately capture disability status. This approach will be instrumental in guiding the development of future health services and policies for people with intellectual disability, as well as improving the alignment of health and disability service provision with the needs of this marginalised group.

### Objectives

This matched retrospective cohort study established one of the largest and most extensive cohorts in the world concerning the health of people with intellectual disability. The cohort was created using multiple linked datasets from New South Wales (NSW), Australia, and serves as an exceptional resource that is representative of Australians with intellectual disability. The availability of comprehensive data spanning a wide array of health and disability services, along with a matched comparator cohort, enables broad analyses of health service utilisation, dispensed medications, healthcare costs, mortality, and associated factors. Our goal is to leverage this resource to inform the development of the sector and services, with a focus on the demands placed on various sector components, variations in prescribing practices, and access to preventive health services. Ultimately, the goal is to improve the health and well-being of individuals with intellectual disability.

The objective of the current cohort profile paper is to provide a detailed description of the cohort’s characteristics, formation, and potential for future research.

## STUDY PARTICIPANTS

The cohort consists of individuals with intellectual disability who have accessed disability and/or health services in NSW, alongside a comparator group of NSW residents without intellectual disability. The comparator group was matched by 5-year age group, sex, and area of residence. Previously, no single data source had systematically collected information on people with intellectual disability in Australia. For cohort formation, we identified individuals with intellectual disability using multiple NSW service datasets. Each dataset adheres to the criteria for a Diagnostic and Statistical Manual of Mental Disorders, fifth edition or International Statistical Classification of Diseases and Related Health Problems, tenth revision (ICD-10) diagnosis of intellectual disability (Table 1). Typically, intellectual disability is characterised by an intelligence quotient of 70 or lower—approximately 2 standard deviations below the mean of 100—and associated impairments in adaptive functioning. Employing multiple datasets to identify people with intellectual disability improves the completeness of capture for this demographic, thereby increasing the probability of obtaining a representative sample across the intellectual disability spectrum. The identifiers used for intellectual disability vary by dataset but are based on robust, formal diagnostic coding practices.

The comparator group consists of NSW residents without intellectual disabilities, who were randomly selected from Medicare Consumer Directory (MCD) records. They were frequency matched to individuals with intellectual disability at a ratio of up to 5:1. The Australian healthcare setting encompasses primary (GP), secondary (specialist) and tertiary (hospital) healthcare services. Medicare, Australia’s national health insurance program, covers the costs of public hospital services. It also subsidises professional health services, such as consultations, surgery, and procedures performed by GPs, specialists, and allied health professionals; pathology tests; medical imaging; eye tests conducted by optometrists; and a very limited range of dental services through the Medicare Benefits Schedule (MBS). Additionally, Medicare subsidises prescribed medications through the Pharmaceutical Benefits Scheme (PBS) for all Australians, permanent residents, and some international visitors. Concession card holders, including pensioners and individuals below a certain income threshold, receive further discounts on dispensed medications. The MCD maintains a record of all individuals registered for Medicare services.

Table 1 summarises all linked datasets included in this study. For the analysis of linked data, the cohort entry date was set as either January 1, 2001 or the date of birth, whichever occurred later, and the analysis period concluded on December 31, 2018 or the date of death, whichever happened earlier.

### Ethics statement

The study was approved by the NSW Population & Health Services Research Ethics Committee (HREC/17/CIPHS/49), the Australian Institute of Health and Welfare (AIHW; EO2017/5/404), ACT Health (ETH.11.17.262), Calvary Public Hospital Bruce (53-2017), Corrective Services NSW (approval date 19/07/2018) and the NSW Department of Education (SERAP2017600).

After record linkage, files containing coded administrative health and other information were uploaded to the Secure Unified Research Environment (SURE) following an established protocol and via a curated gateway. Access to the linked records via SURE is limited to data analysts named in the ethics application. The SURE facility employs a range of information security controls concerning the access, storage and transmission of data. SURE is an accredited platform that undergoes regular audits to ensure it provides a secure environment for data sharing and analysis of sensitive health data. Anonymised data are curated and made available to researchers within a secure setting that meets international standards for data governance and security. Access is granted exclusively to approved researchers, who must use multi-factor authentication, and unit record data cannot be exported from the system. All aggregate statistical outputs undergo a review process to assess any risk of disclosure.

## MEASURES

### Description of the linkage procedure

Administrative databases contain information collected for administrative purposes, such as billing, surveillance, or health and human services monitoring. Data linkage is the process of combining information from different databases that pertains to the same individual. In NSW, the Centre for Health Record Linkage (CheReL) is responsible for the linkage of state administrative datasets. The AIHW Data Linkage Unit carries out the linkage of Commonwealth data. Both agencies maintain secure, high-performance data linkage systems that adhere to the ethical, legal, privacy and confidentiality requirements of the State and Commonwealth. To safeguard the identities and confidentiality of individuals, while still enabling the linking of a person’s data across multiple datasets, each individual is assigned a unique project-specific person number (PPN). Researchers utilise this identifier to merge content data from the linked datasets.

#### Identification of intellectual disability and comparator cohorts

The CHeReL identified individuals with intellectual disability by linking datasets from NSW Disability Services and NSW Health Services, as detailed in Table 1. The Disability Services Minimum Data Set employs the statistical linkage key 581 (SLK581) for individual identification. The SLK581 comprises 4 components: 3 letters from the surname, 2 letters from the given name, date of birth and sex. The distinctiveness of the SLK581 within the Australian population is extremely high, with a uniqueness rate exceeding 98% in the MCD file (regarding correspondence with the AIHW). For privacy preservation, the CHeReL utilised deterministic linkage, which involves exact matching using the SLK581, across all datasets.

To identify comparators, the CHeReL provided the AIHW with the person ID, SLK581, residential postcode, case flag and death flag for all individuals across the NSW datasets over the study period (11.5 million individuals). The AIHW first linked cases to the AIHW National Linkage Map using a dropout-1 linkage approach [31,32], as only the SLK581 was available. This method comprised 11 deterministic passes, with each pass identifying potential record pairs in which all blocking variables matched exactly. Each potential link was assigned a ‘weight’ or ‘comparison score’ based on the level of agreement between records and the uniqueness of information. The AIHW then verified the uniqueness of all accepted record pairs and excluded non-unique matches, which accounted for 1% of the cases. Subsequently, 14% of the cases remained unlinked. These unlinked records were more frequently associated with older individuals and those with unknown sex and birthdate. The AIHW proceeded to identify records with different name codes and/or addresses that corresponded to the same individual. This step uncovered 201 (0.2%) duplicate cases from 951 unique PPNs, and those records were subsequently reassigned to the correct PPNs. Following this deduplication process, the intellectual disability cohort comprised 100,571 cases.

The AIHW identified the comparator cohort from the MCD using the following inclusion criteria: individuals without an intellectual disability, alive as of January 1, 2001, and with a history of residing in NSW. The random selection of comparators was performed using the SAS SURVEYSELECT procedure, with matching based on 5-year age group, sex, and postcode. For the 2,706 linked cases in the NSW cohort that lacked postcode information, the AIHW used the most recent postcode reported on the MCD for matching purposes. Comparators were identified for 99,868 (99%) of the linked cases, resulting in a total comparator cohort of 499,524 individuals.

**Figure 1.**
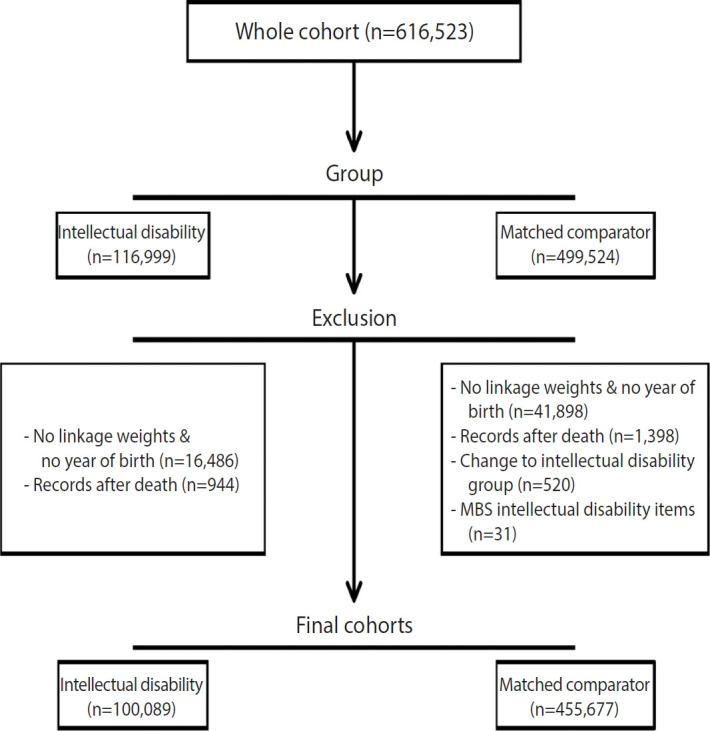
Cohort flowchart. MBS, Medicare Benefits Schedule.

Figure 1 illustrates the composition of the study following the acquisition of data files from the AIHW. The cohort comprised a total of 616,523 unique individuals, including 116,999 people with intellectual disability and 499,524 matched comparators. We excluded 16,486 individuals from the intellectual disability group and 41,898 from the comparator group due to the absence of a linkage ‘weight’ assigned by the AIHW, which is based on the agreement between records and uniqueness of information, or the absence of a recorded year of birth. Additionally, we conducted cross-dataset checks for conflicting information and removed individuals whose date of birth was recorded after their date of death, as well as those with an admission date more than 5 days postmortem. This resulted in the exclusion of 944 individuals from the intellectual disability cohort and 1,398 from the comparator cohort.

In 2015, several supplementary codes for chronic conditions, including disorders of intellectual development and Down syndrome, were introduced into the admitted patient care dataset [33]. These codes had not been previously utilised to define the intellectual disability cohort. Consequently, 520 individuals initially placed in the comparator cohort who had codes for intellectual development disorders or Down syndrome were reassigned to the intellectual disability cohort. Additionally, we excluded 31 individuals (0.01%) from the comparator cohort who had at least 1 record of an intellectual disability-related MBS item number (00718, 00719), which were available from July 2007 to April 2010. Following these adjustments, the intellectual disability cohort comprised 100,089 people, and the comparator cohort consisted of 455,677 individuals (Figure 1).

We will periodically request an update of the linkage when at least 3 years of data have accumulated. As new data become available, we will conduct ongoing checks of hospitalisation and MBS records for the comparator cohort. Consequently, the number of individuals in each group is expected to vary over time.

#### Identification of remoteness area and Index of Relative Socioeconomic Disadvantage

For each cohort member, the AIHW provided the month and year of birth, along with the Statistical Area Level 2 (SA2) code for each recorded residential address. We assigned the 15th of every month as the birth date to estimate age. Since the start and end dates for each residential SA2 were not available, we developed an algorithm to assign a single SA2 to each person. First, we chose the SA2 that appeared most frequently. If the individual had multiple SA2s that appeared with equal frequency, we randomly selected one using a random number generator. We then mapped the SA2 codes from the 2016 version to the Accessibility/Remoteness Index of Australia Plus (ARIA+) and the Index of Relative Socioeconomic Disadvantage (IRSD), utilising data provided by the Australian Bureau of Statistics [34,35]. However, some SA2s did not correspond to the ARIA+ and IRSD indices, leading to missing information for 11% of the intellectual disability cohort and 9% of the comparator group.

### Cohort characteristics

Table 2 presents the demographic profiles of the intellectual disability cohort and the comparator group. Most participants were male (65.3%), and the median age at cohort entry was 3 years (interquartile range [IQR], 0 to 20). The median age at the end of the study period was 21 years (IQR, 14 to 38). Over half of the participants resided in major cities (57.7%), although the cohorts also included individuals from remote and very remote areas (0.9%). Since the cohorts were matched by postcode, comparable proportions lived in the most socioeconomically disadvantaged areas (24.6% of the intellectual disability cohort and 22.6% of the comparator cohort) as well as in the least disadvantaged areas (11.7 and 13.6%, respectively). Approximately two-thirds (68.1%) of the intellectual disability cohort received disability services apart from those for intellectual disability, as recorded in 1 or more of the relevant datasets. Members of the comparator cohort could also receive disability services, provided these services were not for intellectual disability. Within the comparator cohort, a total of 3,477 individuals (0.8%) received such services (Table 2).

### Patient and public involvement

While individuals with disability were not involved in the initial design of the data linkage and cohort study, we plan to actively involve people with intellectual disability (with necessary support), caregivers, and representatives from disability and health service agencies in all subsequent research activities related to this linked dataset. Their contributions will help shape the research direction, assist with the interpretation of preliminary findings, map the potential implications, and develop translational resources associated with the research results. For each research output, we will identify the primary implications for national and jurisdictional health and disability policies and offer specific recommendations to governments and relevant agencies. Additionally, we will outline the implications for services and professionals arising from our research findings. To effectively communicate this information, we will employ a variety of methods: direct engagement with State and Commonwealth health ministries; the preparation of reports for government and policy sectors; peer-reviewed article publication and conference presentations for the scientific community; clinical webinars, online toolkits and practice guides for health professionals; summaries of key findings for clinicians and services; plain English summaries and fact sheets for carers and families; and simplified English and pictorial versions for individuals with intellectual disability.

## KEY FINDINGS TO DATE

In total, the study included 8,538,469 person-years of follow-up: 1,499,236 for people with intellectual disability and 7,039,233 for the comparator cohort.

During the study period, 20,255 deaths occurred (representing 3.7% of the study population): 8,967 (8.9%) among people with intellectual disability and 11,288 (3.4%) among the comparator group. The median age at death for individuals with intellectual disability was 57 years (IQR, 37-70), compared to 69 years (IQR, 55-80) for the comparator group. For demographic information, we calculated frequencies and percentages. To account for differences in follow-up time between the cohorts, we computed person-time for each participant. This allowed us to determine event rates per 1,000 person-years using a Poisson distribution. The overall crude death rate was 6.0 per 1,000 person-years for people with intellectual disability and 2.2 per 1,000 person-years for the comparator cohort.

### Health service use

Table 3 details the health service utilisation by the intellectual disability cohort and the comparator cohort throughout the study period. Most people experienced at least 1 hospitalisation (86.7% in the intellectual disability cohort and 84.9% in the comparator cohort) and at least 1 emergency department visit (85.4 and 87.1%, respectively). Mental health ambulatory services were accessed by 23.3% of the intellectual disability cohort, compared to 8.6% of the comparator cohort. Given the differing mortality rates between the groups, rates of health service utilisation serve as the most informative outcome measures.

Relative to the comparator cohort, people with intellectual disability had double the rate per 100 person-years of all health service contacts.

The cohort with intellectual disability exhibited a higher rate of hospital admissions per 1,000 person-years across all ICD-10 chapters when considering principal diagnosis information. The most prominent disparities between those with intellectual disability and the comparator group were found for dialysis (64 vs. 13 per 1,000 person-years), mental disorders (62 vs. 12 per 1,000 person-years), and diseases of the nervous system and sense organs (42 vs. 16 per 1,000 person-years).

Table 4 presents the numbers and rates per 1,000 person-years for MBS services in the 2 cohorts. Overall, individuals with intellectual disability accessed MBS services at a higher rate per 1,000 person-years (22,225 compared to 13,670). This trend was consistent across all high-level categories of MBS services, except for ‘therapeutic procedures’—including anaesthetics, operations, assistance at operations, obstetrics, and radiotherapy—at 465 versus 522 per 1,000 person-years and oral and maxillofacial services at 2.9 versus 3.6 per 1,000 person-years. The most prominent disparities in MBS service utilisation between the cohorts were seen for cleft lip and cleft palate (2.9 vs. 0.6 per 1,000 person-years) and dental services (93.0 vs. 42.2 per 1,000 person-years).

**Figure 2.**
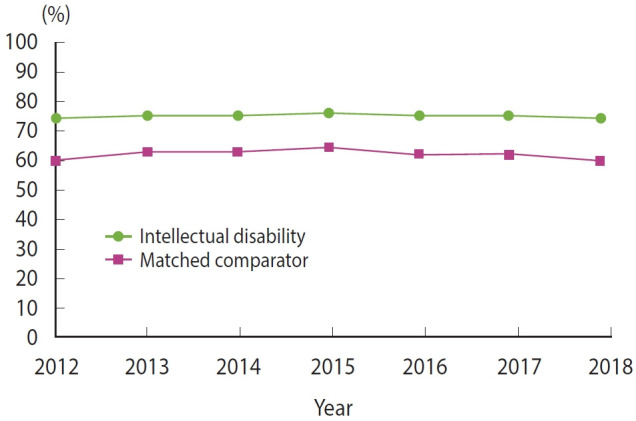
Prescription medicines dispensed to participants with intellectual disability and the comparator cohort from 2012 to 2018.

Before April 2012, the PBS data included only information on PBS medicines dispensed with a government contribution towards the cost. After this date, data collection was expanded to encompass all dispensed PBS medicines, regardless of whether they were given a government subsidy. Concessional beneficiaries—people receiving government benefits, individuals unemployed for extended periods, aged pensioners, and those below a certain income threshold—have a substantially lower co-payment threshold compared to general beneficiaries. Consequently, all medicines dispensed to concessional beneficiaries are subsidised by the government, ensuring complete recording of their medication data. Prior to April 2012, many low-cost medicines were priced below the co-payment level for general beneficiaries, resulting in patients paying the full price. These medicines were not included in the PBS data during that time [36]. Figure 2 shows that after April 2012, approximately 75% of individuals with intellectual disability received PBS-subsidised medicines, compared to about 60% of the comparator cohort.

When comparing dispensed medicines classified by the Anatomical Therapeutic Chemical (ATC) system from July 2012 to December 2018, individuals with intellectual disability were found to have higher rates of medication across all categories (Table 5). On average, individuals with intellectual disability received 15.8 medicines per person per year, in contrast to 6.5 medicines for the comparator cohort. Relative to the comparator cohort, individuals with intellectual disability were prescribed nervous system medications, such as antiepileptics, anti-Parkinson medicines, and antipsychotics, at a rate 4.5 times higher (7.4 vs. 1.6 per person per year). They also exhibited a 2.6-fold higher rate of medicines dispensed for the alimentary tract and metabolism (2.0 vs. 0.8 per person per year).

## STRENGTHS AND WEAKNESSES

One strength of this study is the size of its cohort of individuals with intellectual disability, which constitutes one of the largest cohorts of its kind internationally. NSW is the most populous state in Australia, and demographic similarities across most Australian states and territories facilitate the generalisation of our findings to Australian service users with intellectual disability. However, the results from this cohort study may not be applicable to international populations with different cultural, geographic, and sociodemographic backgrounds, as well as health systems. The study benefits from comprehensive data spanning a wide array of health and disability services, along with a matched comparator cohort. This wealth of information enables extensive analysis of demographic profiles, health service utilisation, dispensed medications, healthcare costs, mortality rates, and associated factors.

A limitation of this study is that administrative data lack detailed clinical information, including potential confounding factors such as disease severity or specific clinical or contextual characteristics, such as the care context. These data also offer limited insight into social factors associated with health status. As such factors are necessarily absent from analytical models, the results require cautious interpretation within this context. In addition, data quality may represent a limitation, as some records may be incomplete or inconsistent. The quality and consistency of the data depend on accurate collection and coding practices. Our cohort of individuals with intellectual disability includes those who have accessed health or disability services; therefore, it is likely that people with mild intellectual disability are under-represented and may have inadvertently been included in the comparator cohort. Moreover, residential location data were not available in chronological order, leading to potential misclassification of residential remoteness and area-based socioeconomic status. However, this misclassification is anticipated to be non-differential. Given our reliance on existing administrative datasets and observational research design, biased estimates of association and residual confounding are possible.

## DATA ACCESSIBILITY

Access to the data and analytical files is restricted to researchers who have been approved by the human research ethics committees, along with data custodians. Researchers interested in collaboration are encouraged to contact the chief investigator at j.trollor@unsw.edu.au with an expression of interest.

## Figures and Tables

**Table 1.** Linked datasets included in this study

Datasets	Description
Admitted Patient Data Collection	Includes admissions to public, private, and multipurpose day hospitals; Records dates of admission and separation for each episode of care, up to 50 diagnoses relevant to each episode, referral source, separation mode, and procedures, based on the Australian version of the ICD-10; Also contains the Australian Diagnosis Related Group and type of admission, which enables estimation of the cost of care; This dataset was used for intellectual disability cohort formation and to identify exposures and outcomes
Australian Immunisation Register	Includes immunisations for children and adults in Australia, as notified by vaccination providers; Records the vaccination type, dose (if applicable), and date
BreastScreen	Details programmatic mammographic screening in women within the recommended age range of 40-74 yr; Records the test date, result, and family history of breast cancer
Cancer Registry	Includes incident cancer diagnoses and cancer-related deaths (notification compulsory); Records cancer type, date of diagnosis, and stage at diagnosis
Cause of Death Unit Record File	Contains coded causes and date of death
Disability Services Minimum Data Set (cohort formation only)	Includes funded disability service recipients in NSW; Records disability type (e.g., autism spectrum disorder and other diagnoses), disability supports including accommodation, community supports, community access and respite, and receipt of specialist programs such as Integrated Services Program and/or Community Justice Program for those with complex needs; This dataset was used for intellectual disability cohort formation and to identify exposures and outcomes
Emergency Department Data Collection	Covers presentations to most emergency departments in public hospitals; Records include dates and times of presentation and discharge, reason for presentation, triage category, and outcome of the presentation (discharge, transfer, or death); This dataset was used for intellectual disability cohort formation and to identify exposures and outcomes
Medicare Benefits Schedule	Primary care in Australia is subsidised through Medicare, a universal healthcare system; Clinicians use the Medicare Benefits Schedule to submit claims for services subsidised by the Australian government; Includes service item number, date, costs, and provider code
Medicare Consumer Directory	Enrolment record of all people registered for Medicare services; This dataset was used for intellectual disability and comparator cohort formation
Mental Health Ambulatory Data Collection	Covers ambulatory mental health services; Records assessment, rehabilitation, or care of non-admitted patients; Ambulatory mental health services are generally those offered to non-admitted patients; These may include mental health day programs, psychiatric outpatient care, and outreach services (e.g., home visits); This dataset was used for intellectual disability cohort formation and to identify exposures and outcomes
National Bowel Cancer Screening Program	Includes all bowel cancer screening and test results for people turning 50, 55, or 65 yr of age; Records the test date and result
National Death Index	Records the fact and coded cause(s) of death registered in Australia
Notifiable Conditions Information Management System	Describes incident infectious conditions requiring compulsory notification including influenza; Records the condition and the date of notification (only for cases in which healthcare was sought)
Ombudsman reviewable deaths (cohort formation only)	Details reviewable deaths in residential care in NSW; Records intellectual disability diagnosis, other disabilities, and the causes and place of death; This dataset was used for intellectual disability cohort formation
Pap Test Register/Cervical Screening	Includes cervical cancer screening test results; Records the test date, type, and result
Pharmaceutical Benefits Schedule	Details all Commonwealth-subsidised medication dispensed, including under co-payment (from 2012 only), and costs
Public Guardian (cohort formation only)	Contains people with disability who received a Public Guardian service in NSW for decision-making assistance in the areas of health and lifestyle; Records intellectual disability diagnoses and guardianship history; This dataset was used for intellectual disability cohort formation
Registry of births, deaths, and marriages – Death Registrations	Includes date of death
State-wide Disability Services (cohort formation only)	Pertains to recipients of disability services in NSW while in custody (a complex needs group); Records the intellectual disability details of offenders; This dataset was used for intellectual disability cohort formation
Targeted specialist support services in public schools (cohort formation only)	Refers to specialist disability services delivered in NSW public schools; These services ensure that the specific needs of students with disability and additional learning and support needs are met, such as through additional staff in the classroom or support for professional learning for teachers; Records intellectual disability, its severity, and the years when students received services; This dataset was used for intellectual disability cohort formation

All datasets include data for people living in NSW and the Australian Capital Territory, apart from datasets utilised solely for cohort formation; The national datasets incorporate records of health services and outcomes for these individuals, irrespective of their place of residence during the follow-up period. ICD-10, International Statistical Classification of Diseases and Related Health Problems, 10th revision; NSW, New South Wales.

**Table 2.** Characteristics of intellectual disability and comparator cohorts in NSW, 2001-2018

Characteristics	Intellectual disability cohort	Comparator cohort
Total	100,089 (18.0)	455,677 (82.0)
Sex		
Female	33,811 (33.8)	159,258 (35.0)
Male	66,278 (66.2)	296,419 (65.0)
Age at cohort entry (yr)^1^		
Median (IQR)	3 (0-19)	4 (0-20)
Mean±SD	12±18	13±18
Age at end of study period (yr)		
Median (IQR)	21 (14-37)	22 (14-38)
Mean±SD	27±19	28±19
Age at death (yr)		
Median (IQR)	57 (37-70)	69 (55-80)
Mean±SD	52±24	64±22
Remoteness^2^		
Major city	57,831 (57.8)	260,972 (57.3)
Inner regional	22,850 (22.8)	111,104 (24.4)
Outer regional	7,347 (7.3)	38,691 (8.5)
Remote	382 (0.4)	1,888 (0.4)
Very remote	420 (0.4)	1,950 (0.4)
Missing	11,259 (11.3)	41,102 (9.0)
Index of relative socioeconomic disadvantage^3^		
1-2 (most disadvantaged)	24,666 (24.6)	102,787 (22.6)
3-4	22,085 (22.1)	99,440 (21.8)
5-6	21,173 (21.1)	97,890 (21.5)
7-8	15,391 (15.4)	78,622 (17.3)
9-10 (least disadvantaged)	11,673 (11.7)	62,173 (13.6)
Missing	5,101 (5.1)	14,765 (3.2)
Appeared in disability services datasets unrelated to intellectual disability		
No	31,900 (31.9)	452,200 (99.2)
Yes	68,189 (68.1)	3,477 (0.8)

Values are presented as number (%). NSW, New South Wales; IQR, interquartile range; SD, standard deviation.

1 Age at cohort entry was calculated as the difference between year of birth and 2001; for those born in 2001 or later, the age at cohort entry was set to zero.

2 Statistical Area 2 was mapped to the Mesh block, which was then mapped to the remoteness area [35].

3 Statistical Area 2 was used to assign an area-based index of relative socioeconomic disadvantage [34].

**Table 3.** Health service utilisation by intellectual disability and comparator cohorts in NSW, 2001-2018

Variables	Intellectual disability cohort	Comparator cohort
Total	100,089 (18.0)	455,677 (82.0)
Mental health ambulatory treatment days		
No. of people with at least 1 treatment day	23,368 (23.3)	39,252 (8.6)
Female	7,913 (23.4)	15,049 (9.4)
Male	15,455 (23.3)	24,203 (8.2)
Rate per 1,000 PY (95% CI)	1,012 (1,011, 1,014)	157 (157, 158)
ED presentations		
No. of people with at least 1 ED presentation	85,465 (85.4)	397,007 (87.1)
Female	28,537 (84.4)	129,901 (81.5)
Male	56,928 (86.0)	267,106 (90.1)
Rate per 1,000 PY (95% CI)	707 (706, 709)	379 (379, 380)
Hospitalisations		
No. of people with at least 1 hospital episode	86,823 (86.7)	387,057 (84.9)
Female	29,785 (88.1)	137,775 (86.5)
Male	57,038 (86.5)	253,916 (85.7)
Rate per 1,000 PY (95% CI)	538 (537, 539)	235 (235, 235)
Rate of hospital admissions per 1,000 PY		
Other factors influencing health	44	33
Maternal, neonatal, and congenital causes	38	26
Digestive system diseases	45	23
Injury and poisoning	40	23
Respiratory diseases	42	19
Dialysis	64	13
Mental disorders	62	12
Nervous and sense disorders	42	16
Symptoms and abnormal findings	16	10
Genitourinary diseases	12	11
Musculoskeletal diseases	14	7
Infectious diseases	10	7
Circulatory diseases	7	6
Neoplasms-malignant	10	5
Skin diseases	11	3
Endocrine diseases	3	3
Neoplasms-other than malignant	7	2
Blood and immune diseases	4	1
No diagnosis	44	33
Total (95% CI)	538 (537, 539)	235 (235, 235)

Values are presented as number (%). NSW, New South Wales; PY, person-years; CI, confidence interval; ED, emergency department.

**Table 4.** Number and rate per 1,000 person-years of MBS services utilised by intellectual disability and comparator cohorts in NSW, 2001-2018

MBS category and broad type of service	Intellectual disability cohort		Comparator cohort	
	n (%)	Rate	n (%)	Rate
Professional attendances	13,906,808 (41.7)	9,275.90	41,646,906 (43.3)	5,916.40
Non-referred attendances-Enhanced primary care	832,337 (2.5)	555.2	1,469,578 (1.5)	208.8
Non-referred attendances-Other	457,468 (1.4)	305.1	1,586,054 (1.6)	225.3
Non-referred attendances GP/Vocationally registered GP	9,653,062 (29.0)	6,438.7	31,779,432 (33.0)	4,514.6
Optometry	330,674 (1.0)	220.6	1,635,735 (1.7)	232.4
Specialist attendances	2,633,267 (7.9)	1,756.4	5,176,107 (5.4)	735.3
Diagnostic procedures and investigations	388,641 (1.2)	259.2	1,122,237 (1.2)	159.4
Therapeutic procedures	696,936 (2.1)	464.9	3,677,396 (3.8)	522.4
Anaesthetics	92,204 (0.3)	61.5	490,173 (0.5)	69.6
Assistance at operations	5,883 (0.0)	3.9	66,410 (0.1)	9.4
Obstetrics	25,601 (0.1)	17.1	426,751 (0.4)	60.6
Operations	282,075 (0.8)	188.1	1,655,688 (1.7)	235.2
Other MBS services	186,603 (0.6)	124.5	432,350 (0.4)	62.6
Radiotherapy and therapeutic nuclear medicine	23,289 (0.1)	15.5	197,322 (0.2)	28.0
Specialist attendances	81,281 (0.2)	54.2	408,702 (0.4)	61.4
Oral and maxillofacial services	4,296 (0.0)	2.9	25,165 (0.0)	3.6
Diagnostic imaging services	1,108,519 (3.3)	739.4	4,652,626 (4.8)	661.0
Pathology services	7,530,342 (22.6)	5,022.8	24,312,188 (25.3)	3,453.8
Pathology collection items	2,868,081 (8.6)	1,913.0	9,657,281 (10.0)	1,371.9
Pathology tests	4,662,261 (14.0)	3,109.8	14,654,907 (15.2)	2,081.9
Cleft lip and cleft palate services	4,337 (0.0)	2.9	4,379 (0.0)	0.6
Dental services	139,382 (0.4)	93.0	297,170 (0.3)	42.2
Miscellaneous services	9,541,196 (28.6)	6,364.0	20,493,287 (21.3)	2,911.3
Non-referred attendances-Practice nurse	234,339 (0.7)	156.3	677,228 (0.7)	96.2
Other allied health	981,211 (2.9)	654.5	1,450,932 (1.5)	206.1
Other MBS services	8,325,646 (25.0)	5,553.3	18,365,127 (19.1)	2,609.0
Total MBS services	33,320,457 (100)	22,225.0	96,231,354 (100)	13,670.7

MBS, Medicare Benefits Schedule; NSW, New South Wales; GP, general practitioner.

**Table 5.** Number and rate per 1,000 person-years of dispensed medicines by ATC classification for intellectual disability and comparator cohorts in NSW, July 2012-December 2018

Variables	Intellectual disability cohort		Comparator cohort	
	n (%)	Rate	n (%)	Rate
Alimentary tract and metabolism	1,191,576 (12.7)	2,013.0	2,223,898 (11.9)	779.9
Blood and blood forming organs	153,833 (1.6)	259.9	477,400 (2.5)	167.4
Cardiovascular system	1,133,926 (12.1)	1,915.6	4,249,763 (22.7)	1,490.3
Dermatologicals	148,502 (1.6)	250.9	434,801 (2.3)	152.5
Genitourinary system and sex hormones	190,488 (2.0)	321.8	506,938 (2.7)	177.8
Systemic hormonal preparations, excluding sex hormones and insulins	184,640 (2.0)	311.9	489,149 (2.6)	171.5
Anti-infectives for systemic use	928,549 (9.9)	1,568.6	2,993,650 (16.0)	1,049.8
Antineoplastic and immunomodulating agents	86,158 (0.9)	145.5	221,572 (1.2)	77.7
Musculo-skeletal system	230,391 (2.5)	389.2	628,316 (3.4)	220.3
Nervous system	4,370,325 (46.6)	7,382.9	4,636,065 (24.7)	1,625.8
Antiparasitic products, insecticides and repellents	9,339 (0.1)	15.8	20,317 (0.1)	7.1
Respiratory system	466,726 (5.0)	788.5	1,242,002 (6.6)	435.6
Sensory organs	234,577 (2.5)	396.3	552,169 (2.9)	193.6
Various	32,759 (0.3)	55.3	30,387 (0.2)	10.7
Other	9,856 (0.1)	16.6	14,490 (0.1)	5.1
No ATC information	3,379 (0.0)	5.7	21,579 (0.1)	7.6
Total	399,353 (100)	15,837.4	1,556,931 (100)	6,572.7

ATC, Anatomical Therapeutic Chemical; NSW, New South Wales.

## References

[1] American Psychiatric Association (APA) (2013). Diagnostic and statistical manual of mental disorders.

[2] Maulik PK, Mascarenhas MN, Mathers CD, Dua T, Saxena S (2011). Prevalence of intellectual disability: a meta-analysis of population-based studies. Res Dev Disabil.

[3] Cooper SA, McLean G, Guthrie B, McConnachie A, Mercer S, Sullivan F (2015). Multiple physical and mental health comorbidity in adults with intellectual disabilities: population-based cross-sectional analysis. BMC Fam Pract.

[4] Liao P, Vajdic C, Trollor J, Reppermund S (2021). Prevalence and incidence of physical health conditions in people with intellectual disability - a systematic review. PLoS One.

[5] Tuffrey-Wijne I, Goulding L, Giatras N, Abraham E, Gillard S, White S (2014). The barriers to and enablers of providing reasonably adjusted health services to people with intellectual disabilities in acute hospitals: evidence from a mixed-methods study. BMJ Open.

[6] Buckley N, Glasson EJ, Chen W, Epstein A, Leonard H, Skoss R (2020). Prevalence estimates of mental health problems in children and adolescents with intellectual disability: a systematic review and meta-analysis. Aust N Z J Psychiatry.

[7] Mazza MG, Rossetti A, Crespi G, Clerici M (2020). Prevalence of co-occurring psychiatric disorders in adults and adolescents with intellectual disability: a systematic review and meta-analysis. J Appl Res Intellect Disabil.

[8] Burke E, O’Dwyer M, Maes-Festen D, Oppewal A, Sheerin F, Doyle C (2023). Intellectual disabilities: health and social care across the lifespan.

[9] Hirvikoski T, Boman M, Tideman M, Lichtenstein P, Butwicka A (2021). Association of intellectual disability with all-cause and cause-specific mortality in Sweden. JAMA Netw Open.

[10] Trollor J, Srasuebkul P, Xu H, Howlett S (2017). Cause of death and potentially avoidable deaths in Australian adults with intellectual disability using retrospective linked data. BMJ Open.

[11] Florio T, Trollor J (2015). Mortality among a cohort of persons with an intellectual disability in New South Wales, Australia. J Appl Res Intellect Disabil.

[12] Glover G, Williams R, Heslop P, Oyinlola J, Grey J (2017). Mortality in people with intellectual disabilities in England. J Intellect Disabil Res.

[13] Ouellette-Kuntz H, Shooshtari S, Balogh R, Martens P (2015). Understanding information about mortality among people with intellectual and developmental disabilities in Canada. J Appl Res Intellect Disabil.

[14] Tyrer F, Kiani R, Rutherford MJ (2020). Mortality, predictors and causes among people with intellectual disabilities: a systematic narrative review supplemented by machine learning. J Intellect Dev Disabil.

[15] Mann C, Jun GT, Tyrer F, Kiani R, Lewin G, Gangadharan SK (2023). A scoping review of clusters of multiple long-term conditions in people with intellectual disabilities and factors impacting on outcomes for this patient group. J Intellect Disabil.

[16] Lonchampt S, Gerber F, Aubry JM, Desmeules J, Kosel M, Besson M (2021). Prevalence of polypharmacy and inappropriate medication in adults with intellectual disabilities in a hospital setting in Switzerland. Front Psychiatry.

[17] Haider SI, Ansari Z, Vaughan L, Matters H, Emerson E (2014). Prevalence and factors associated with polypharmacy in Victorian adults with intellectual disability. Res Dev Disabil.

[18] O’Dwyer M, Peklar J, McCallion P, McCarron M, Henman MC (2016). Factors associated with polypharmacy and excessive polypharmacy in older people with intellectual disability differ from the general population: a cross-sectional observational nationwide study. BMJ Open.

[19] Lunsky Y, Elserafi J (2012). Antipsychotic medication prescription patterns in adults with developmental disabilities who have experienced psychiatric crisis. Res Dev Disabil.

[20] Sheehan R, Hassiotis A, Walters K, Osborn D, Strydom A, Horsfall L (2015). Mental illness, challenging behaviour, and psychotropic drug prescribing in people with intellectual disability: UK population based cohort study. BMJ.

[21] Tyrer P, Cooper SA, Hassiotis A (2014). Drug treatments in people with intellectual disability and challenging behaviour. BMJ.

[22] Odalović M, Gorman A, Paul A, McCallion P, Burke É, MacLachlan M (2024). Psychotropic medicines’ prevalence, patterns and effects on cognitive and physical function in older adults with intellectual disability in Ireland: longitudinal cohort study, 2009-2020. BJPsych Open.

[23] Saari K, Koponen H, Laitinen J, Jokelainen J, Lauren L, Isohanni M (2004). Hyperlipidemia in persons using antipsychotic medication: a general population-based birth cohort study. J Clin Psychiatry.

[24] Lennox NG, Green M, Diggens J, Ugoni A (2001). Audit and comprehensive health assessment programme in the primary healthcare of adults with intellectual disability: a pilot study. J Intellect Disabil Res.

[25] Weise J, Pollack A, Britt H, Trollor JN (2017). Primary health care for people with an intellectual disability: an exploration of consultations, problems identified, and their management in Australia. J Intellect Disabil Res.

[26] Cocks E, Thomson A, Thoresen S, Parsons R, Rosenwax L (2016). Health status and use of medications by adults with intellectual disability in Western Australia. J Intellect Dev Disabil.

[27] Haveman M, Perry J, Salvador-Carulla L, Walsh PN, Kerr M, Van Schrojenstein Lantman-de Valk H (2011). Ageing and health status in adults with intellectual disabilities: results of the European POMONA II study. J Intellect Dev Disabil.

[28] Hermans H, Evenhuis HM (2014). Multimorbidity in older adults with intellectual disabilities. Res Dev Disabil.

[29] Reppermund S, Heintze T, Srasuebkul P, Reeve R, Dean K, Smith M (2019). Health and wellbeing of people with intellectual disability in New South Wales, Australia: a data linkage cohort. BMJ Open.

[30] Reppermund S, Srasuebkul P, Heintze T, Reeve R, Dean K, Emerson E (2017). Cohort profile: a data linkage cohort to examine health service profiles of people with intellectual disability in New South Wales, Australia. BMJ Open.

[31] https://www.aihw.gov.au/getmedia/18e1666a-8b29-44d7-86d8-65bc1f6e2cb5/dlpuslk.pdf.aspx?inline=true.

[32] Karmel R, Anderson P, Gibson D, Peut A, Duckett S, Wells Y (2010). Empirical aspects of record linkage across multiple data sets using statistical linkage keys: the experience of the PIAC cohort study. BMC Health Serv Res.

[33] https://www.aihw.gov.au/reports/chronic-disease/supplementary-codesfor-chronic-conditions/summary.

[34] https://www.abs.gov.au/ausstats/abs@.nsf/Lookup/by%20Subject/2033.0.55.001~2016~Main%20Features~SOCIO-ECONOMIC%20INDEXES%20FOR%20AREAS%20(SEIFA)%202016~1.

[35] https://www.abs.gov.au/ausstats/abs@.nsf/mf/1270.0.55.001.

[36] Mellish L, Karanges EA, Litchfield MJ, Schaffer AL, Blanch B, Daniels BJ (2015). The Australian Pharmaceutical Benefits Scheme data collection: a practical guide for researchers. BMC Res Notes.

